# Metabolome Based Reaction Graphs of *M. tuberculosis* and *M. leprae*: A Comparative Network Analysis

**DOI:** 10.1371/journal.pone.0000881

**Published:** 2007-09-12

**Authors:** Ketki D. Verkhedkar, Karthik Raman, Nagasuma R. Chandra, Saraswathi Vishveshwara

**Affiliations:** 1 Molecular Biophysics Unit, Indian Institute of Science, Bangalore, India; 2 Bioinformatics Centre, Supercomputer Education and Research Centre, Indian Institute of Science, Bangalore, India; The Wellcome Trust Sanger Institute, United Kingdom

## Abstract

**Background:**

Several types of networks, such as transcriptional, metabolic or protein-protein interaction networks of various organisms have been constructed, that have provided a variety of insights into metabolism and regulation. Here, we seek to exploit the reaction-based networks of three organisms for comparative genomics. We use concepts from spectral graph theory to systematically determine how differences in basic metabolism of organisms are reflected at the systems level and in the overall topological structures of their metabolic networks.

**Methodology/Principal Findings:**

Metabolome-based reaction networks of *Mycobacterium tuberculosis*, *Mycobacterium leprae* and *Escherichia coli* have been constructed based on the KEGG LIGAND database, followed by graph spectral analysis of the network to identify hubs as well as the sub-clustering of reactions. The shortest and alternate paths in the reaction networks have also been examined. Sub-cluster profiling demonstrates that reactions of the mycolic acid pathway in mycobacteria form a tightly connected sub-cluster. Identification of hubs reveals reactions involving glutamate to be central to mycobacterial metabolism, and pyruvate to be at the centre of the *E. coli* metabolome. The analysis of shortest paths between reactions has revealed several paths that are shorter than well established pathways.

**Conclusions:**

We conclude that severe downsizing of the leprae genome has not significantly altered the global structure of its reaction network but has reduced the total number of alternate paths between its reactions while keeping the shortest paths between them intact. The hubs in the mycobacterial networks that are absent in the human metabolome can be explored as potential drug targets. This work demonstrates the usefulness of constructing metabolome based networks of organisms and the feasibility of their analyses through graph spectral methods. The insights obtained from such studies provide a broad overview of the similarities and differences between organisms, taking comparative genomics studies to a higher dimension.

## Introduction

Recent advances in high throughput technologies and network theory have made it possible to reconstruct and analyse large genome-scale networks of organisms *in silico.* Several types of networks reflecting different aspects of metabolism and regulation in organisms have been reconstructed. The transcriptional networks based on microarray data, protein-protein interaction networks based on high-throughput yeast two-hybrid type of experiments and metabolic networks based on reaction annotation of the individual proteins coded by the genome are some examples. Several of these studies have focused on elucidating the general principles underlying the structure and organisation of metabolic networks of a large number of organisms. For example, a protein–protein interaction network of *Saccharomyces cerevisiae* constructed based on systematic two-hybrid analyses [1] indicates that highly connected proteins with a central role in the network's architecture are three times more likely to be essential than proteins with only a small number of links to other proteins [2]. Similarly, a transcriptional regulatory network of *Escherichia coli*, has been reconstructed [3] based on the RegulonDB database as well as published literature, and has been used to identify important structural network motifs and their role in network function. The dynamics of the *Saccharomyces cerevisiae* biological network has been investigated computationally, with the integration of transcriptional regulatory information and gene-expression data for multiple conditions [4]. Metabolic networks have also been constructed for a number of genomes such as *E. coli* [5] and *Staphylococcus aureus* [6] which have been used to study the metabolic capabilities of organisms and gene essentiality through flux balance analyses. Protein-protein interaction networks have been previously used for comparative genomics [7], [8], [9], [10], [11]. Here, we seek to exploit the reaction-based networks of three organisms for comparative genomics. We use concepts from spectral graph theory to systematically determine how differences in the basic metabolism of various organisms are reflected at the systems level.

In the present study, we have constructed and characterised the metabolic networks of two closely related organisms: *Mycobacterium tuberculosis* and *Mycobacterium leprae*, which are obligate intracellular pathogens [12]. A broad comparison with the network of *E. coli* has also been presented. Both the mycobacteria are important pathogens and hence of interest. Additionally, their comparison is of particular interest given that the genome sequencing of *M. leprae* has revealed massive gene decay as compared to other mycobacteria. Despite having genomes of comparable sizes the leprae genome codes for only 1406 proteins in comparison to the 3989 proteins in *M. tuberculosis*, leading to the consideration of the leprae genome as a ‘minimal genome’. The elimination of many important metabolic activities in this organism is thought to result in severe metabolic streamlining [12].

Various concepts from graph theory [13], [14], [15], [16], [17] have previously been used to construct and analyse metabolic networks for several fully sequenced organisms. Representing metabolic networks as graphs makes them amenable to various analyses, such as the detection of shortest and alternate paths. Such analyses have also resulted in the identification of the highly connected giant strong components of the networks, as well as metabolites central to the network. Graph spectral analysis can be carried out to obtain information on central hubs as well as the sub-clustering and organisation of the metabolic network.

We represent the metabolic networks of these organisms in the form of a reaction based graph with the biochemical reactions as the nodes and an edge existing between the nodes if they share at least one metabolite. Such a representation is essential to ascertain the importance of different biochemical reactions in the metabolic networks of these organisms. Alternative representations have been previously used for representing metabolite networks. One is a substrate graph, wherein all substrates are represented by nodes, with edges between them indicating their participation in the same reaction [18]. In another representation, the metabolic network is built up of nodes, the substrates, which are connected to one another through links, which are the actual metabolic reactions. The physical entity of the link is the temporary educt–educt complex itself, in which enzymes provide the catalytic scaffolds for the reactions yielding products, which in turn can become educts for subsequent reactions [13]. Metabolic networks may also be represented as a directed bipartite graph, with two types of nodes indicating reactions and metabolites separately, with edges from metabolites directed towards the reaction they are substrates of and edges from reactions directed towards their products [19].

Further, in our representation of the network, we have chosen to leave out the links generated by currency metabolites. These metabolites are ubiquitously present in metabolic networks and although these substrates are necessary for a given reaction to take place, they cannot be considered as valid intermediates for path finding or establishing biologically meaningful network connections. Currency metabolites have been excluded from metabolic networks in previous studies such as [17].

Besides characterising the constructed networks for their various graph properties, we have systematically determined if the differences observed between these organisms at the genomic level reflect on the overall topology and characteristics of their metabolic networks. To compare features of the metabolic networks of mycobacteria with those of a more standard and well studied organism, we have also constructed and characterised the metabolic network of *E. coli*.

Metabolic networks have properties similar to other real world networks, such as social networks and the World Wide Web. Particularly, they have been shown to be scale-free, with a non-random power-law distribution of node connectivity (number of interactions of each metabolite) and distinguished by the presence of ‘hubs’, a few highly connected nodes that are essential to the integrity and robustness of the network [13]. These networks are also small-world networks, characterised by a low average path length between nodes [13], [17], [18], [20]. Recent studies have revealed metabolic networks to be modular in nature, comprising several small, functional modules that combine together in a hierarchical manner to form larger, less cohesive units [21], [22].

In this work, we have studied the topological organisation of the constructed metabolic networks by using concepts from spectral graph theory. The graph spectral method has been applied earlier by our group in the identification of side chain and backbone clusters in proteins and to identify amino acid residues important for protein structure, folding, stability, function and dynamics [23], [24]. It has also been used to successfully identify domains in multi-domain proteins [25] and to detect clusters of structurally similar proteins in protein chain universe graphs [26]. Bu and co-workers [27] have applied spectral analysis to study the topological structure of the protein interaction network in yeast. Jernigan and co-workers [28] have used spectral graph theory for analysing the functional clustering of the yeast protein–protein interaction network. Here, we explore the applications of the spectral method in analysing the topological organisation of large reaction networks. We show that this method is useful for identifying sub-clusters of reactions in the networks by a simple one step numerical computation.

## Results and Discussion

Several pathway databases have recently become available, with curated information on biochemical reactions. Most databases are incomplete, with missing information on various biochemical reactions. A study undertaken recently by Kettner [29] illustrates the poor quality in well-known databases such as BRENDA even for pathways such as glycolysis. They conclude from their study that the difficulty in curation and enhancing quality in databases was largely due to both incomplete descriptions of material and methods in the papers and difficulties considering method-dependent results, since extraction of kinetic data from literature is necessarily carried out manually. They suggest that it might be useful to establish a deposition system to which authors can submit their data to ensure maximal accuracy and accessibility and to replace possibly the traditional retrospective process of manual data extraction. The situation is a bit better when it comes to reaction annotation without the quantitative data, for obvious reasons. At this point of time, there does not appear to be an automated method for detecting and correcting errors. Although highly desirable, availability of a comprehensive accurate database, hence may be quite a while away. We have used KEGG as our primary source of data, since it was the largest available curated database, particularly for mycobacteria. A major problem we observed was in describing the reversibility of the reactions, which we have corrected manually to the extent possible. Although there are omissions and errors in detail in the database, on the whole, most of the reactions and proteins are annotated correctly, from the manual checks we performed. Hence, the KEGG serves as a good starting point for systems biology studies. KEGG has also been extensively used by several groups previously for systems analyses [16], [17], [30], [31] and even genome-scale metabolic reconstructions [32]. Felix and Valiente have performed an exhaustive validation of a substantial portion of the KEGG LIGAND database [33], concluding that over 90% of the reactions in the KEGG are consistent.

The reaction networks of *M. tuberculosis* H37Rv, *M. leprae* and *E. coli* K-12 MG1655 were reconstructed from a dataset primarily obtained from the KEGG LIGAND database [34]. We used reaction files containing compound IDs, so that there was no discrepancy in the reactions, based on the usage of synonymous compound names. Details of the total numbers of reactions, metabolites and enzymes comprising the networks of these organisms have been summarised in Table 1. The total size of the networks in the three organisms is roughly of the same order, making their comparison quite meaningful. The networks essentially capture the core metabolic processes, such as the metabolism of carbohydrates, nucleic acids, amino acids and lipids, in the three organisms. Sequence analyses of the genomes have indicated that they share similarity among a number of individual genes/proteins, leading to several similar reactions in the three networks. However, the precise connections with which the reactions are associated have not been analysed previously in a comparative context. Graphs presented here are amenable to such comparative analysis, providing a handle to understand the similarities and the differences between a given pair of organisms from a systems perspective. Analysis of the various network properties, clustering patterns, shortest and alternate paths, that aid in this process are elaborated below.

**Table 1:** Network properties of the reconstructed metabolic networks of *M. tuberculosis*, *M. leprae* and *E. coli* and the corresponding random networks.

Property	*M. tuberculosis*	*M. leprae*	*E. coli*
**DETAILS OF THE RECONSTRUCTED NETWORK**			
Total no. of reactions (nodes)	1906	1325	2080
Total no. of edges	14,100	8,508	20,316
Reversible reactions	209	152	274
Irreversible reactions	1488	1021	1532
No. of metabolites participating in the reactions	1649	1139	1633
Total no. of proteins catalyzing the reactions	1097	469	1062
No. of currency metabolites eliminated	102	84	107
**NETWORK PARAMETERS**			
Percentage of nodes belonging to the largest cluster	73.40	76.15	73.85
Percentage of ‘orphan’ nodes	17.00	16.30	16.39
Highest degree of connections	72	50	96
Average path length	5.58	5.48	4.94
Clustering co-efficient	0.01669	0.01600	0.01614
Degree exponent of the power law degree distribution	1.3952	1.4423	1.4093
**PROPERTIES OF RANDOM NETWORKS** *			
Percentage of nodes belonging to the largest cluster	99.94	99.85	99.99
Percentage of ‘orphan’ nodes	0.0588	0.1487	0.0058
Highest degree of connections	22	21	27
Average path length	4.00±0.02	4.07±0.03	3.61±0.01
Clustering co-efficient	(3.78±0.56)*10^−3^	(4.84±0.89)*10^−3^	(4.67±0.45)*10^−3^
Characteristic Scale	7.40±0.0122	6.42±0.0137	9.76±0.0134

* Random networks have been generated using the Erdős-Rényi model, with the same total number of vertices and edge probability as the corresponding reaction networks.

### Analysis of network parameters

**Figure 1:**
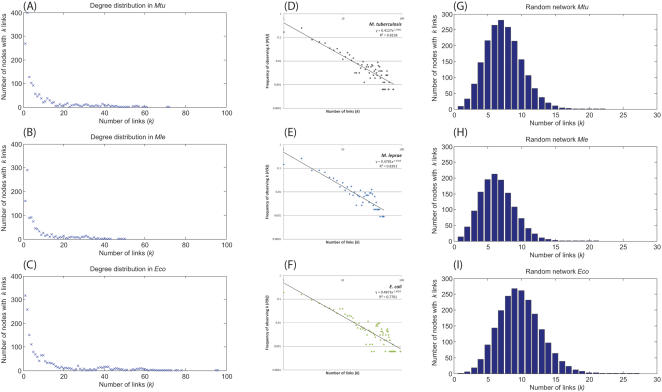
A–C: Plots of the degree distributions of nodes in the reaction networks of *M. tuberculosis*, *M. leprae* and *E. coli.* D–F: Log-log plots of the degree distribution function *P(k)* versus the degree *k*. *P(k)* defines the probability of a given node making exactly *k* connections in the network. The fit to the curve shows a power law behaviour and has an exponent of ∼1.4 for all three networks. G–I: Representative degree distributions of nodes in random networks generated with the same total number of nodes and edges as the reaction networks of *M. tuberculosis*, *M. leprae* and *E. coli.*

Analysis of various network parameters such as the degree distribution, clustering coefficient, average path length and size of the largest cluster of the reaction networks of all three organisms revealed them to have a similar overall topology with comparable graph properties (Table 1). The degree distributions of the nodes in the reaction networks of all three organisms exhibit a power law behaviour as shown in Figures 1A−C. Hence, the reaction networks of these organisms are scale-free in nature, consisting of a few ‘hubs’ that are highly connected and hold together numerous nodes having a small degree. The log-log plots of the degree distributions (Figures 1D–F) for the three networks also are characteristic of a power law behaviour, with the degree exponent γ∼1.4. This result differs from the results of Wagner and Fell [18] who did not obtain a clear power-law degree distribution for a reaction based graph of the metabolic network of *E. coli.* This was perhaps due to the reduced dataset used in their study. The clustering coefficients of the reaction networks of the three organisms are of the same order of 10^−2^, indicating that these networks are quite sparse and have approximately the same density of connections between nodes despite a difference in their overall sizes. Further, it implies that the observed connections may have evolved to suit a particular metabolic requirement and are far from random. The average path length of the three networks are also comparable, with the networks of *M. tuberculosis* and *M. leprae* having average path lengths of 5.58 and 5.48 respectively, while the reaction network of *E. coli* has a slightly smaller average path length of 4.94. The small values of the average path length in these organisms imply a small-world character, where the distance between any two reactions in the network is smaller than what is expected from traditional biochemical pathways, as will be shown later.

### Clustering patterns

**Figure 2:**
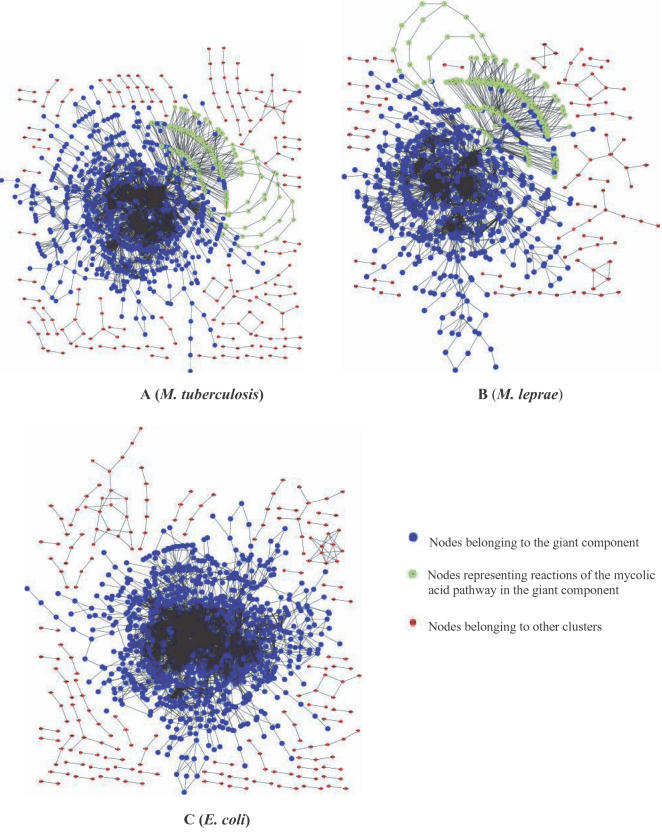
Clusters in the reaction networks of *M. tuberculosis* (A), *M. leprae* (B) and *E. coli* (C). The giant component in the networks comprises 73–76% of the total number of nodes, while 8–10% of the total nodes form other clusters in the networks. The orphan nodes have been eliminated for better visualisation.

The largest clusters (giant components) in the networks of *M. tuberculosis*, *M. leprae* and *E. coli* were obtained by the depth-first-search method and comprised 73–76% of the total number of biochemical reactions in their metabolome; while 8–10% of the total reactions form other clusters in their respective networks. It is therefore interesting to note that the size of the giant component in the reaction networks of these organisms is conserved and is unaffected by the metabolic streamlining of the leprae bacillus. It is also unaffected by differences that exist between the metabolism of *E. coli* and the mycobacterial organisms. Figures 2A–C are representations of the clusters obtained in the reaction networks of the three organisms. Graphviz [35] was used for the generation of the cluster diagrams. Clustering analysis of the three networks by the depth-first-search method also revealed several ‘orphan’ reactions that were completely unconnected in their respective networks. Some of these were reactions that involved only currency metabolites and hence were unconnected with the rest of the network due to our elimination of interactions mediated by the currency metabolites. Other orphans represented reactions which were either inaccurately curated in the database and did not occur in the metabolism of these organisms or whose links with the rest of the metabolome have not been determined to date. A few examples of orphan reactions are shown below:

**R00004 (EC 3.6.1.1.) Pyrophosphate+H2O→2 Orthophosphate**

**R00281 (EC 1.6.99.3) Acceptor+NADH→Reduced-acceptor+NAD+**

As an extreme example, we even have:

**R02501 (EC 1.14.14.1) Testosterone+H++Oxygen+NADPH→19-Hydroxytestosterone+NADP^+^**

Such information on orphan reactions can be used to improve the curation of the database and the annotation of the genome itself.

We compared the network properties of the three reaction networks with those of random graphs comprising the same total number of vertices (V) and connecting edges (E) as the corresponding reaction network, using the Erdős-Rényi model (see Table 1). It was observed that for the given probability of connections, the nodes of the random network formed a single connected cluster comprising almost 100% of the nodes. Further, the degree distributions of the nodes of the random networks having the same total number of (V, E) as that of the *M. tuberculosis*, *M. leprae* and *E. coli* reaction networks followed a Poisson distribution, with an average scale of 7.40, 6.42 and 9.76 connections per node respectively. Moreover, the highest degree of connections in these networks was significantly lower with values of 22, 21 and 27 as compared to the highest degree in the reaction networks of *M. tuberculosis* (72), *M. leprae* (50) and *E. coli* (96) respectively.

### Analyses of sub-clusters in the giant component

Metabolic networks are highly integrated and complex in nature. Hence, a rational reduction of these networks to their basic structural and functional units is essential to gain a deeper understanding of their design principles and functioning. Several studies have been carried out to detect modularity in metabolic networks [21], [22], [36], [37]. Guimera & Nunes Amaral [38] have shown that metabolites in the cell group together to form functional modules with typically 80% of the nodes in the network only connected to other nodes within their respective modules. To determine if this modularity of metabolic networks is also reflected at the level of the constituent biochemical reactions, we carried out analyses to detect sub-clusters of reactions in the giant component by graph spectral analysis.

**Figure 3:**
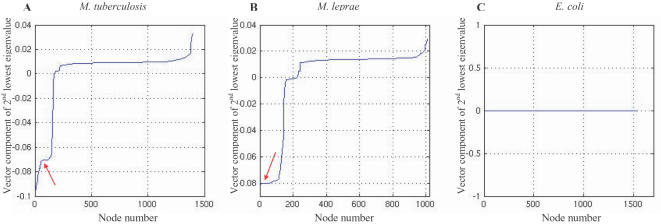
2evc plots for the giant components of the reaction networks of *M. tuberculosis* (A), *M. leprae* (B) and *E. coli* (C). Plateaus represent sub-clusters of reactions. The giant component in the reaction network of *E. coli* does not resolve into sub-clusters as indicated by the single plateau in plot C. Arrows indicate plateaus representing the sub-cluster of mycolic acid pathway reactions in the mycobacterial networks.

As mentioned in the methods section, the 2evc plots of the Laplacian matrix of the graph provide the sub-cluster information. The 2evc plots constructed for the reaction networks of *M. tuberculosis* and *M. leprae* comprised numerous plateau regions which represented sub-clusters of reactions in the giant component of these networks (Figures 3A,B). A few examples of the sub-clusters obtained in the giant component of *M. tuberculosis* have been shown in Table 2.

**Table 2:** Examples of sub-clusters in the giant component of the metabolic network of *M. tuberculosis*.

2evc	Node no.	RID*	Pathway-ID	Pathway
CLUSTER 1		(SIZE: 12)		
0.00902	552	R01700	map00020	Citrate cycle (TCA cycle)
0.00902	1129	R04231	map00770	Pantothenate and CoA biosynthesis
0.00902	1130	R04233	rn00770	Pantothenate and CoA biosynthesis
0.00902	181	R00439B	rn00230	Purine metabolism
0.00902	168	R00429	rn00230	Purine metabolism
0.00902	278	R00722B	rn00230	Purine metabolism
0.00902	173	R00434	rn00230	Purine metabolism
0.00902	118	R00332B	rn00230	Purine metabolism
0.00902	164	R00416B	map00530	Aminosugars metabolism
0.00902	331	R00896	rn00272	Cysteine metabolism
0.00902	200	R00480	rn00260	Gly, Ser and Thr metabolism
0.00902	443	R01214F	rn00280	Val, Leu, Ile degradation
CLUSTER 2		(SIZE: 11)		
0.00921	483	R01343F	map00220	Urea cycle and metabolism of amino groups
0.00921	453	R01248F	rn00220	Urea cycle and metabolism of amino groups
0.00921	455	R01251F	rn00220	Urea cycle and metabolism of amino groups
0.00921	999	R03646B	rn00330	Arg and Pro metabolism
0.00921	457	R01253	rn00330	Arg and Pro metabolism
0.00921	834	R02788	rn00300	Lys biosynthesis
0.00921	135	R00376F	rn00230	Purine metabolism
0.00921	612	R01857B	rn00230	Purine metabolism
0.00921	701	R02235F	rn00790	Folate biosynthesis
0.00921	703	R02236F	rn00670	One carbon pool by folate
0.00921	1542	R05688	rn00100	Biosynthesis of steroids
CLUSTER 3		(SIZE: 10)		
0.00675	1204	R04536B	rn00061	Fatty acid biosynthesis
0.00675	1206	R04543B	rn00061	Fatty acid biosynthesis
0.00675	1343	R04958B	rn00061	Fatty acid biosynthesis
0.00675	1346	R04961B	rn00061	Fatty acid biosynthesis
0.00675	1349	R04964B	rn00061	Fatty acid biosynthesis
0.00675	1351	R04966B	rn00061	Fatty acid biosynthesis
0.00675	1340	R04955B	rn00061	Fatty acid biosynthesis
0.00675	1180	R04429B	rn00061	Fatty acid biosynthesis
0.00675	1202	R04534B	rn00061	Fatty acid biosynthesis
0.00675	1338	R04953B	rn00061	Fatty acid biosynthesis
CLUSTER 4		(SIZE:24)		
−0.07038	1810	MAP123	fasII	Mycolic acid pathway
−0.07038	1812	MAP125	fasII	Mycolic acid pathway
−0.07038	1813	MAP126	fasII	Mycolic acid pathway
−0.07038	1814	MAP127	fasII	Mycolic acid pathway
−0.07038	1816	MAP129	fasII	Mycolic acid pathway
−0.07038	1794	MAP107	fasII	Mycolic acid pathway
−0.07038	1818	MAP131	fasII	Mycolic acid pathway
−0.07038	1819	MAP132	fasII	Mycolic acid pathway
−0.07038	1820	MAP133	fasII	Mycolic acid pathway
−0.07038	1822	MAP135	fasII	Mycolic acid pathway
−0.07038	1824	MAP137	fasII	Mycolic acid pathway
	.			
	.			
	.			
−0.07038	1825	MAP138	fasII	Mycolic acid pathway
−0.07038	1826	MAP139	fasII	Mycolic acid pathway
−0.07038	1795	MAP108	fasII	Mycolic acid pathway

* In the RID, F indicates the reaction proceeding in the forward direction, and B indicates the reaction proceeding in the backward direction.

It is observed that reactions belonging to fatty acid biosynthesis and the FAS-II cycle of the mycolic acid pathway in *M. tuberculosis* form distinct, tightly connected sub-clusters. This may be due to the iterative nature of these cycles where the metabolite passes through several cycles of the same reactions consecutively in order to obtain a product of the requisite carbon chain length. Hence, the reactions within these pathways are more tightly connected with each other than to the other reactions in the metabolome. The mycolic acid pathway [39], [40], [41] is a critical pathway in *M. tuberculosis* and is important for its survival and pathogenicity. The other sub-clusters obtained are less specific and contain reactions belonging to different pathways identified in the KEGG. For example, cluster 1 in Table 2 comprises reactions involved in purine metabolism as well in pantothenate and coenzyme-A biosynthesis. Further, reactions occurring in a particular pathway are not contained in a single cluster but different reactions from the same pathway are observed in different sub-clusters. For example, reactions involved in purine metabolism occur in both clusters 1 and 2.

The reaction network of *M. leprae* comprised sub-clusters that were fewer in number and smaller in size than those obtained in *M. tuberculosis.* This is because the metabolome of *M. leprae* comprises fewer reactions than that of *M. tuberculosis.* Analysis of these sub-clusters revealed them to be similar in nature to those of *M. tuberculosis*, with reactions of the FAS-II cycle of the mycolic acid pathway forming closely related sub-clusters while the other sub-clusters were less specific in nature. Therefore, the overall topological structure and nature of the giant component is conserved between the two mycobacteria, indicating that the large scale downsizing of the *M. leprae* metabolome has not significantly altered the global structure of its core reaction network. Interestingly, unlike the mycobacterial networks, the giant component in the reaction network of *E. coli* did not resolve into constituent sub-clusters (Figure 3C), implying that reactions occurring in the metabolome of *E. coli* are more strongly interconnected across various biochemical pathways. Thus, differences can be seen to exist in the finer grouping of biochemical reactions within the giant components of the reaction networks of the mycobacteria and *E. coli*.

Lastly, from the analyses of sub-clusters in the giant components of the three reaction networks, it can be seen that functional modules are less well defined at the level of biochemical reactions, with the reactions forming a single, large, connected cluster ensuring free flow of metabolites between them. However, it is important to note that similar to other studies, our model also is limited by the assumption of constitutive expression of all enzymes catalysing these reactions, i.e. the temporal expression of the enzymes on account of regulation is ignored. This may affect the clustering of biochemical reactions.

### Identification of hubs in the reaction networks

Metabolic networks are scale-free networks characterised by the presence of hubs that are highly connected nodes serving to hold together numerous smaller nodes having a lower degree [13]. With a large number of links, hubs in metabolic networks integrate all substrates in the cell into a single, complex web of biochemical interactions. Hubs are essential to the integrity and robustness of the network against random attacks [42], [43]. They are also responsible for the small-world behaviour of networks as any two nodes in the network can be reached by a relatively short distance by traversing a hub [43]. Furthermore, in biological networks, the hubs are thought to be functionally important and phylogenetically oldest [18], [20], [21], [44]. To identify highly connected reactions essential and central to the metabolism of the three organisms under study, we elucidated the hubs in their reaction networks by graph spectral analysis as well as by degree analysis.

As mentioned in the methods section, the largest vector component of the highest eigenvalue of the Laplacian matrix of the graph corresponds to the node with high degree as well as low eccentricity. Two parameters, degree and eccentricity, are involved in the identification of graph spectral (GS) hubs. In a graph representing a scale-free network, the highest vector component would therefore correspond to a hub with high degree and also closest to the geometric centre of the network. Alternatively, hubs can be ranked based on their connectivity alone (degree hubs). Thus, hubs obtained from graph spectral analysis may differ from the degree hubs. However, a comparison of the ranks of GS hubs and degree hubs did not show any significant difference (Supplementary Table S1). This is perhaps due to the topology of the network.

However, when the reactions corresponding to the hubs were examined in detail, we find discrimination between the two sets of hubs. It was observed that the top 50 degree hubs in the reaction networks of the three organisms comprised reactions involving the metabolite L-glutamate as well as reactions involving pyruvate. However, the top 50 GS hubs of *M. tuberculosis* and *M. leprae* exclusively comprised reactions involving L-glutamate while the top GS hubs in *E. coli* only consisted of reactions involving pyruvate (see Supplementary Table S2). The difference in the degree and GS hubs suggests that the most highly connected reactions are not necessarily the most central reactions in the metabolome of the organism (by centrality, we mean that node which has the least eccentricity; eccentricity is the distance between a node and the node farthest from it). Furthermore, reactions (and metabolites) forming the centres of reaction networks are not common across all organisms but are specific to the metabolism of each organism. Previous studies by Ma & Zeng [17] on a substrate based metabolic network of *E. coli* have shown pyruvate to be central to the metabolism of this organism; our results corroborate their observation. By constructing a reaction based graph of the metabolic network, we have elucidated specific reactions involving pyruvate that form the centre of the *E. coli* metabolome. Moreover, our method of identifying hubs central to the network by graph spectral analysis is simpler and more advantageous as it uses a single numerical computation that takes into account both the degree and eccentricity of the hub in the overall network.

The quality of the initial database used to reconstruct the network would affect the results obtained in this analysis. An incomplete database may lead to several false positives in the identification of hubs in the network. To determine the reliability of the identified hubs, we randomly knocked out half the nodes in the original reaction networks of the three organisms to generate highly reduced networks that simulate an incompletely curated database. This exercise was repeated 30 times for each organism and it was observed that reactions involving L-glutamate comprised a majority of the top 20 GS hubs in the reduced *M. tuberculosis* and *M. leprae* networks 83% and 67% of the time respectively. Similarly reactions involving pyruvate formed the majority of the top 20 GS hubs in the reduced *E. coli* network 67% of the time. Hence, we believe that the results of our analysis presented here are fairly reliable.

### Identification of mycobacterial hubs absent in the human metabolome

Hubs are essential to the integrity of the network. They make the network vulnerable to targeted attacks on them, because once the highly connected hubs are attacked, the network starts disintegrating [42], [43]. This property makes the hubs ideal drug targets; by targeting a drug against a suitable hub or group of hubs, it is possible to break down the cellular network of an organism completely, which would result in the death of the organism. Further, targeting hubs that are unique to the pathogen and absent in humans would minimise side-effects of the drug in the host. We analysed the GS hubs in *M. tuberculosis* and *M. leprae* to identify highly connected reactions that are central to the metabolism of these organisms but absent in humans (as detailed in the Methods section). The enzymes catalysing these reactions can be explored further as potential drug targets in the field of knowledge-based, rational drug design. The top fifteen ‘unique’ hubs obtained in *M. tuberculosis* (Table 3) mainly comprise reactions involved in nitrogen metabolism and in the biosynthesis of essential amino acids that are not synthesised in humans. It is particularly interesting to note that the first reaction of the FAS-I cycle of the mycolic acid pathway unique to mycobacteria ranks as the 38^th^ unique hub in mycobacterium. Thus, the enzyme acyl carrier protein-fatty acid synthase (ACP-FAS) involved in this reaction can be explored as a potential target for drugs against mycobacteria. Independent studies by Sassetti and co-workers [45] and Raman and co-workers [41] have also identified the FAS enzyme as one of the putative anti-tubercular drug targets. Sassetti and co-workers performed a high throughput Transposon Site Hybridisation Mutagenesis study to identify essential genes in *M. tuberculosis* while Raman and co-workers performed a flux balance analysis of the mycolic acid pathway in *M. tuberculosis*, followed by *in silico* gene deletions to identify essential genes/putative drug targets. The list of the top hubs unique to *M. leprae* is similar and has not been tabulated separately.

**Table 3:** Top fifteen hubs in *M. tuberculosis* that are absent in the metabolome of humans.

Rank as a unique hub	Rank as a GS hub	Node no.	Hub RID	E.C. No. of catalyzing enzyme	Name of Enzyme	Pathway(s) for which the enzyme is required
1	3	368	R00986	4.1.3.27	Anthranilate synthase	Phenylalanine, tyrosine and tryptophan biosynthesis
2	4	482	R01339F	2.6.1.-	Transferases, transaminases	Nitrogen metabolism
3	8	1197	R04475B	2.6.1.17	Succinyldiaminopimelate aminotransferase	Lysine biosynthesis
4	12	197	R00457B	2.6.1.36	Transferases, Transaminases	Lysine biosynthesis
5	14	717	R02283B	2.6.1.11	Acetylornithine transaminase	Urea cycle and metabolism of amino groups
6	28	160	R00411	3.5.1.-	Hydrolases acting on carbon-nitrogen bonds, other than peptide bonds in linear amides	Arginine and proline metabolism
7	35	714	R02282f	2.3.1.35	Glutamate N-acetyltransferase	Urea cycle and metabolism of amino groups
8	48	19	R00114	1.4.1.13	L-glutamate synthase	Glutamate metabolism, Nitrogen metabolism
9	49	562	R01716	6.3.5.8	Aminodeoxychorismate synthase	Folate biosynthesis
10	54	87	R00260F	5.1.1.3	Glutamate racemase	Glutamate metabolism, D-Glutamine and D-glutamate metabolism
11	55	1440	R05225	6.3.5.10	Cobyric acid synthase	Porphyrin and chlorophyll metabolism
12	64	1439	R05224	6.3.5.9	Hydrogenobyrinic acid a,c-diamide synthase (glutamine-hydrolysing)	Porphyrin and chlorophyll metabolism
13	83	1707	R06860	2.5.1.64	2-succinyl-6-hydroxy-2,4-cyclohexadiene	Ubiquinone biosynthesis
14	85	1458	R05320	1.14.11.17	Taurine dioxygenase	Taurine and hypotaurine metabolism
15	86	442	R01197	1.2.7.3	2-oxoglutarate synthase	Citrate cycle
.						
.						
38*	172	1692	MAP005	-	Acyl carrier protein-fatty acid synthase I (ACP-FAS)	Mycolic acid pathway

* ACP-FAS enzyme involved in the fifth reaction of the mycolic acid pathway ranks as the 38^th^ unique hub in *M. tuberculosis.*

### Analysis of shortest paths

Metabolic networks are small-world networks characterised by a small average path length between the nodes. Thus, in the metabolic network of most organisms, on an average, any metabolite can be converted to any other metabolite by rather small number of biochemical reactions [13]. Besides analysing the reaction networks of the three organisms under study to determine their average path lengths as described in a previous section, we have also elucidated the actual steps in the shortest path between any two nodes in their networks. We compared the shortest paths obtained in these organisms across six different metabolic pathways: (a) glycolysis, (b) citric acid cycle, (c) pentose phosphate pathway, (d) phenylalanine, tyrosine and tryptophan biosynthesis, (e) valine, leucine and isoleucine biosynthesis and (f) folate biosynthesis and observed that a majority of the paths obtained between reactions from these pathways were the same in all three organisms. Hence, the streamlining of the *M. leprae* metabolome has not significantly altered the shortest routes that exist between reactions in its core metabolism. Further, the shortest paths between reactions of the above pathways are also unaffected by the significant differences in the metabolism of mycobacteria and *E. coli.*

As predicted from the low values of the average path length for these reaction networks, the paths obtained in this analysis are shorter than the traditionally annotated biochemical pathways. For example, the conversion of pyruvate to oxalosuccinate by the experimentally determined citric acid cycle requires a minimum of four steps (pyruvate→oxaloacetate→citrate→isocitrate→oxalosuccinate) while the shortest path obtained between them by our analysis comprised only three consecutive steps:

Steps in terms of RIDs:

**R00344F→R00355B→R00268b**

**R00344 (EC 6.4.1.1) ATP+Pyruvate+HCO3−→ADP+Orthophosphate+Oxaloacetate**

**R00355 (EC 2.6.1.1) Oxaloacetate+L-Glutamate→L-Aspartate+2-Oxoglutarate**

**R00268 (EC 1.1.1.42) 2-Oxoglutarate+CO2→Oxalosuccinate**

Hence, it can be inferred that the traditionally annotated pathways are not the shortest possible routes for the conversion of one metabolite into another. They are therefore a result of several constraints imposed within the cell, such as intracellular compartmentalisation and thermodynamic constraints. Whether the shortest pathways predicted here are really feasible pathways or not should be evaluated in the context of the constraints in the cell.

Interestingly, our analysis also revealed shortest paths in which one or more reactions in the path produce metabolites that are utilised by reactions other than those that would be involved in the commonly accepted notion of the steps of a given pathway. Though such paths do not represent an ideal shortest path comprising consecutive steps between a pair of reactions (as in the above example), it provides important information regarding the interaction of reactions from different biochemical pathways. For example, the set of reactions obtained as the shortest path between reaction R00959 (the 1^st^ reaction) and R00658 (the 8^th^ reaction) in the glycolytic pathway are indicated in Table 4.

**Table 4:** Shortest path between steps in glycolytic pathway.

Steps in terms of RIDs:	
R00959F→R02740F→R01830F→R01826F→R00658F	
R00959 (EC 5.4.2.2)	D-Glucose-1-phosphate→alpha-D-Glucose-6-phosphate
R02740 (EC 5.3.1.9)	alpha-D-Glucose-6-phosphate→beta-D-Fructose-6-phosphate
R01830 (EC 2.2.1.1)	beta-D-Fructose-6-phosphate+(2R)-2-Hydroxy-3-(phosphonooxy)-propanal→D-Erythrose-4-phosphate+D-Xylulose-5-phosphate
R01826 (EC 2.5.1.54)	Phosphoenolpyruvate+D-Erythrose-4-phosphate+H2O→2-Dehydro-3-deoxy-D-arabino-heptonate-7-phosphate+Orthophosphate
R00658 (EC 4.2.1.11)	2-Phospho-D-glycerate→Phosphoenolpyruvate+H2O

The path comprises reactions from different metabolic pathways – reaction R02740 occurs in the glycolysis and pentose phosphate pathways, reaction R01830 occurs in the pentose phosphate pathway and reaction R01826 occurs in the phenylalanine, tyrosine and tryptophan biosynthetic pathway. Further, in reaction R01826 phosphoenolpyruvate produced in the glycolytic pathway reacts with D-erythrose-4-phosphate produced in the pentose phosphate pathway to yield 2-dehydro-3-deoxy-D-arabino-heptonate-7-phosphate, a precursor for tryptophan biosynthesis. It is clear that the flux through each of these reactions will influence the direction of metabolites into specific biochemical pathways. For example, the phosphoenolpyruvate produced in reaction R00658 can either be converted into pyruvate by the glycolytic route or be diverted into tryptophan biosynthesis.

The results of this analysis are also likely to be useful for metabolic engineering. A systematic study of shortest paths between all reaction pairs in the metabolome of an organism can reveal paths that are shorter than traditionally annotated biochemical pathways. Understanding of such paths can have implications in the production of industrially important secondary metabolites and for the manipulation of particular reaction fluxes to direct intermediates into specific metabolic pathways for obtaining larger quantities of the desired product.

### Analysis of alternate paths

We have extensively analysed the number of alternate paths, that exist between reaction pairs in the metabolomes of the three organisms (for this, all 312 reactions common to the three organisms were considered). It was observed that *E. coli* contained the most number of alternate paths between a given pair of reactions, followed by *M. tuberculosis*, while the leprae bacillus had the least number of alternate paths of the three organisms. This is due not only to the differing sizes of the networks of the three organisms, but also to the difference in the degree distributions. Though this result is not surprising in itself, it is interesting to note that the reductive evolution of the *M. leprae* metabolome has led to a loss of multiple paths between reactions pairs in the core metabolic pathways, while keeping the shortest possible route between them intact. This is possibly because only the core of the metabolome has been conserved; the alternate routes (in *M. tuberculosis*) add to the redundancy. Similarly, mycobacteria and *E. coli* have evolved to have differing number of alternate paths between reactions while keeping the shortest paths between the reactions conserved.

### Conclusions

The results presented in this work show that the reaction networks of *M. tuberculosis*, *M. leprae* and *E. coli* are scale-free, small-world networks that differ significantly from random networks. The networks of the three organisms have similar network properties despite a difference in the overall sizes of their metabolomes.

Graph spectral theory serves as a tool useful for analysing the topological structure and organisation of large complex networks. This technique yields information about the sub-clustering of nodes in the network and identifies the cluster centres by a single numeric computation. Analysis of sub-clusters of the mycobacterial reaction networks detected by this method suggests that modularity of metabolic networks is possibly less well-defined at the level of biochemical reactions; clusters have been discerned well from metabolite networks [21]. It was observed that the top 50 GS hubs of *M. tuberculosis* and *M. leprae* exclusively comprised reactions involving L-glutamate while the top GS hubs in *E. coli* only consisted of reactions involving pyruvate. We showed that the most highly connected biochemical reactions in the reaction network of an organism are not necessarily the reactions most central to the metabolism of that organism. Moreover, the reactions and metabolites forming the centre of the metabolic networks are not common across all organisms, but are specific to the metabolism of each organism.

A systematic comparison of the topological properties of the mycobacterial metabolic networks reveals that massive gene decay in *M. leprae* does not significantly affect the global structure of its metabolic network. We showed that the most highly connected reactions central to the metabolism of both organisms as well as the finer grouping of reactions within the giant component of their networks are essentially conserved and differ from that of *E. coli*. Additionally, we have determined that metabolic streamlining in the leprae bacillus has led to the preservation of the shortest paths between reactions in its core metabolome while reducing the total number of alternate paths that exist between them. The results obtained in this work are also useful likely to find applications in rational drug design and metabolic engineering.

## Methods

### Dataset

We used the KEGG LIGAND database to reconstruct the reaction networks of *M. tuberculosis* H37Rv, *M. leprae* and *E. coli* K-12 MG1655 from genome data. A list of the metabolic pathways and their constituent biochemical reactions in these organisms were downloaded as flat files. These files contain information about the reactants, products, reversibility and steady state stoichiometries of the biochemical reactions. We corrected these files for several mistakes such as incomplete reactions and inconsistencies in reaction reversibility and direction. Polymerisation reactions were eliminated from these files and not included in the network construction. The KEGG database does not contain complete information on the mycolic acid pathway (MAP), which is unique to mycobacteria and essential for their pathogenicity and survival. Hence, to obtain more complete and organism-specific models of the reaction networks of *M. tuberculosis* and *M. leprae,* we integrated the model of the MAP developed by Raman and co-workers [41] into the corrected flat files of these organisms. The MAP model was integrated *in toto* for *M. tuberculosis,* while 13 reactions involved in extending the products of the FAS–II cycle of MAP into methoxy-mycolates were not included in the *M. leprae* model as methoxy-mycolates are absent in the leprae cell wall [12].

### Metabolic network reconstruction

The metabolic networks of the three organisms were constructed as follows. Each biochemical reaction in the metabolome of the organism is a node, and nodes representing reactions that share a common metabolite as a substrate in one reaction and a product in the other are connected by an edge. Thus, only reactions which exhibit such a consecutive dependence on each other with respect to a metabolite are connected to each other. All edges have an equal weight of 1. In order to make the network amenable to numerical analysis, it is represented in the form of an adjacency or reaction-interaction matrix (RIM), which is an *n*×*n* matrix; *n* being the number of nodes (biochemical reactions) in the graph. The elements [*A*]*ij* of the RIM have values according to the following rules:

[*A*]*ij* = 1 if reactions *i* and *j* exhibit a consecutive dependence through a metabolite

 = 0 if the reactions do not exhibit such a dependence, or if *i* = *j*

To construct the RIM, the set of reactions in the flat file representing the metabolome of each organism was first mathematically represented as a stoichiometric matrix, **S** *_m_*_×*n*_, with every metabolite being represented by a row (*m* metabolites) and every reaction by a column (*n* reactions). The entries in each column correspond to the stoichiometric coefficients of the metabolites (negative for reactants and positive for products) for each reaction. The *i^th^* row of the matrix defines the participation of a particular metabolite across all metabolic reactions, and the *j^th^* column provides the stoichiometry of all metabolites in that reaction. In order to account for the reversibility of biochemical reactions, each reversible reaction was split up into two irreversible reactions with opposite signs of the stoichiometric coefficients in the stoichiometric matrix. Further, to remove interactions between reactions by virtue of ubiquitously present ‘currency metabolites’ such as ATP, ADP, NADP, H_2_O, CO_2_, Coenzyme-A etc., matrix rows representing currency metabolites were discarded. The complete list of currency metabolites discarded is given in Supplementary Text S1.

To derive the RIM from the stoichiometric matrix, consecutive dependences between pairs of reactions were determined. This was done by comparing the signs of the stoichiometric coefficients of each metabolite participating in the reactions between the two columns representing the reaction pair in the stoichiometric matrix. A (+,−) or (−,+) relationship between the coefficients in row *m* of the stoichiometric matrix compared across column *i* and *j* indicated a consecutive dependence between the *i^th^* and *j^th^* reaction with respect to metabolite *m* and the element [A]*ij* in the corresponding RIM was assigned the value 1. The RIMs thus constructed are symmetric and served as the primary input for the various analyses carried out in this study. The construction of the stoichiometric matrix and the corresponding RIM for 4 hypothetical reactions R1–R4 has been pictorially represented in Supplementary Figure S1.

### Graph Spectral Analysis

Graph spectral theory is a sub-field of graph theory that deals with the analysis of the spectra (eigenvalues and eigenvector components) of nodes in the graph. Such an analysis provides information on the overall structure and topology of the graph. To obtain the eigenvalue spectra of the graph, the adjacency matrix of the graph is converted to a Laplacian matrix (**L**), by the equation:
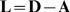
where **D**, the degree matrix of the graph, is a diagonal matrix in which the *i^th^* element on the diagonal is equal to the number of connections that the *i^th^* node makes in the graph. Diagonalisation of the Laplacian matrix yields the spectra of the graph comprising the eigenvalues and their corresponding eigenvector components. Analysis of the vector components of the lower and higher eigenvalues yields information about the clustering of nodes in the graph and the connectivity of each node in the graph [23], [46]. Specifically, the vector components of the second lowest eigenvalue carry information about the clusters present in the graph with all nodes of a given cluster having the *same* value of the vector component [47]. In a completely connected graph, where all nodes belong to a single cluster, the vector components of the second lowest eigenvalue provide sub-cluster information where the nodes within a sub-cluster have similar vector component values. A sub-cluster is a set of nodes within a cluster that make significantly more connections among themselves than with the other nodes in the cluster. A plot of the sorted vector components of the second lowest eigenvalue (2evc) versus the node number clearly reveals the sub-cluster information, with the nodes belonging to a sub-cluster forming distinct plateaus on the curve and the nodes connecting the sub-clusters having vector component values in between the plateaus. Identification of sub-clusters by the graph spectral method has been applied successfully in various studies such as the identification of domains in multi-domain proteins [25] and to detect clusters of structurally similar proteins in protein chain universe graphs [26]. The vector components of the top eigenvalues contain information regarding the branching of nodes in the graph and the nodes forming the cluster centres [23], [48]. The largest vector component of the highest eigenvalue corresponds to the node with the highest degree in the graph. However, in case there are many nodes with degenerate highest degree, the one which is closest to the geometric centre of the graph, takes up the highest vector component value. Similarly, the second largest vector component of the highest eigenvalue corresponds to the node with next highest degree and next lowest eccentricity in the graph and so on. Eccentricity, *E(v)*, of a vertex *v* in a graph **G** is the distance from *v* to the vertex farthest from *v* in G, i.e.
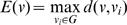
where *d*(*v*,*v_i_*) represents the length of the shortest path between *v* and *v_i_*.

### Identification of mycobacterial hubs absent in the human metabolome

To identify hubs in the reaction networks of *M. tuberculosis* and *M. leprae* that were absent in the metabolism of humans, we first obtained a comprehensive list of biochemical reactions occurring in the metabolome of *Homo sapiens* from the KEGG database. Each reaction in this database is identified with a unique reaction-id (RID) that is common across all organisms in the database. Further, each RID is associated with the E.C. number of the enzyme catalysing the reaction which is further linked with the RIDs of the other reactions catalysed by the enzyme. For each reaction in the list of hubs for the two organisms, we obtained the E.C. number and associated RIDs of its corresponding enzyme. These RIDs, including the RID of the primary hub reaction were compared with the list of RIDs of reactions occurring in humans. An absence of all the RIDs in humans implied that the particular enzyme did not function in the metabolome of humans and the primary hub reaction was then classified as a hub ‘unique’ to the mycobacterium. However, since the data on the metabolism of humans was obtained from the KEGG database, it is important to note that the verity of the unique hub depends on the quality of the database.

### Analysis of shortest and alternate paths

The shortest paths in the network and the steps involved were identified using the Floyd-Warshall algorithm [49]. For the analysis of alternate paths, the adjacency matrix was used as the primary input. The total number of alternate paths between any two nodes in the network was determined by using classical algorithms that count paths based on powers of matrices [50]. Essentially, by this method, the value of the element [*A*]*ij* of the matrix [*A*] obtained by raising the adjacency matrix to the power *n* is equal to the total number of paths of path length *n* that exist between node *i* and node *j* in the graph.

## Supporting Information

Table S1 Comparison of top 50 graph spectral and degree hubs in mycobacteria
(0.03 MB XLS)

Table S2 Reactions corresponding to the top 20 GS and degree hubs in the three organisms
(0.01 MB TXT)

Text S1 Currency metabolites in the three organisms
(0.01 MB TXT)

Figure S1 Construction of stoichiometric matrix and reaction influence matrix for a hypothetical set of reactions
(0.03 MB DOC)
