# Supplementary material for: Metabolome Based Reaction Graphs of M. tuberculosis and M. leprae: A Comparative Network Analysis
Source: PLoS One. 2007 Sep 12;2(9):e881. doi: 10.1371/journal.pone.0000881 (PMC1964534; doi:10.1371/journal.pone.0000881)
Supplement: Figure S1 — Construction of stoichiometric matrix and reaction influence matrix for a hypothetical set of reactions (0.03 MB DOC) [file pone.0000881.s004.doc]

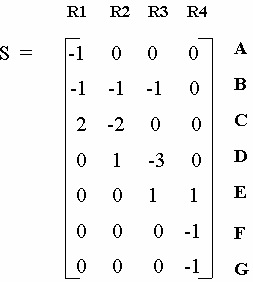


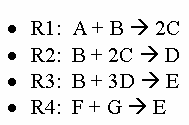


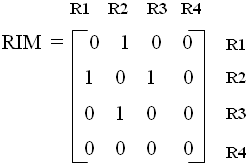


**Supplementary Figure S1:** The stoichiometric matrix (S) and reaction-interaction matrix (RIM) constructed for 4 hypothetical reactions R1-R4. A-G represent metabolites participating in the 4 reactions. The reactions that exhibit a consecutive dependence on each other for one or more metabolites are connected by an edge in the RIM. The construction of the stoichiometric matrix and the derivation of the RIM is described in detail in the Methods section.
